# Drug repurposing of sophoridine for sepsis-induced organ injury: from in-depth analysis of a single agent to a multi-target therapeutic paradigm

**DOI:** 10.3389/fphar.2026.1800840

**Published:** 2026-06-24

**Authors:** Hongxiao Ma, Cheng Peng, Xiaonan Liu, Xinyu Zhang, Mingduan Sun, Mingyi Lü

**Affiliations:** 1 Affiliated Zhongshan Hospital of Dalian University, Dalian, Liaoning, China; 2 Hongqi Hospital Affiliated to Mudanjiang Medical University, Mudanjiang, Heilongjiang, China

**Keywords:** drug delivery system, drug repurposing, inflammatory, network pharmacology, organ dysfunction, precision medicine, sepsis, sophoridine

## Abstract

Sepsis-induced immune dysregulation and multiple organ dysfunction present formidable clinical challenges that conventional single-target therapies fail to address. This review evaluates the potential of the natural quinolizidine alkaloid sophoridine for drug repurposing in sepsis. Pharmacological evidence demonstrates that sophoridine exerts multi-target synergistic effects by modulating the nuclear factor-κB (NF-κB) signaling pathway, the NOD-like receptor family pyrin domain-containing 3 (NLRP3) inflammasome, and programmed cell death pathways. To overcome translational hurdles such as temporal progression and microenvironmental heterogeneity, we propose a precision therapeutic framework. This strategy integrates biomarker-driven dynamic dosing, nanotechnology-enabled targeted delivery, and multi-omics-guided systems pharmacology. Ultimately, this paradigm aims to provide a rigorous theoretical basis for developing personalized, spatiotemporally controlled interventions for sepsis-associated organ injury.

## 1 Introduction

While the inflammatory cascade and consequent tissue injury in sepsis remain central therapeutic challenges, current interventions largely fail to halt these underlying pathological processes. In this context, drug repurposing has emerged as a promising strategy for innovative sepsis therapy, offering established pharmacokinetic profiles and accelerated translational potential. Sophoridine is a natural quinolizidine alkaloid extracted mainly from the roots of *Sophora flavescens* Aiton (Fabaceae). It has a documented history of more than 2,000 years in traditional Chinese medicine (TCM) theory and clinical practice. As a key bioactive constituent of total alkaloids from *S. flavescens*, sophoridine exhibits remarkable potential for multi-target and multi-dimensional intervention against sepsis: pharmacokinetic studies confirm its favorable tissue distribution and relatively long elimination half-life *in vivo*; Furthermore, pharmacological evidence demonstrates that sophoridine exerts robust anti-inflammatory, antioxidant, and immunomodulatory effects. It achieves these therapeutic benefits by synergistically regulating multiple pivotal signaling cascades. Key targets include the nuclear factor-κB (NF-κB) pathway, the NOD-like receptor family pyrin domain-containing 3 (NLRP3) inflammasome, the nuclear factor erythroid 2-related factor 2/antioxidant response element (Nrf2/ARE) axis, and the Janus kinase/signal transducer and activator of transcription (JAK-STAT) pathway. These pleiotropic pharmacological effects align well with the complex pathophysiological network of sepsis.

**FIGURE 1:**
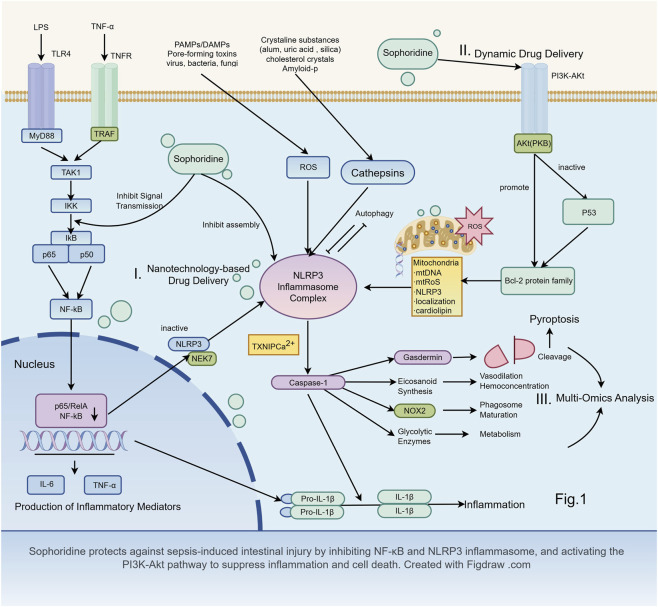
The multi-target regulatory network and therapeutic paradigm of sophoridine in sepsis. Sophoridine intervenes in sepsis-induced organ injury by modulating key inflammatory and apoptotic pathways. (I) Nanotechnology-based drug delivery ensures targeted accumulation in inflammatory sites. (II) Dynamic drug delivery precisely matches the temporal progression of sepsis. (III) Multi-omics analysis elucidates the underlying pharmacological mechanisms. Solid arrows indicate activation or promotion, while blunt-ended lines indicate inhibition. Abbreviations: NF-κB, nuclear factor-κB; NLRP3, NOD-like receptor family pyrin domain-containing 3; ROS, reactive oxygen species.

Notably, with the iterative advancement of delivery technologies—from conventional oral or intravenous administration to intelligent drug-loading systems such as nanoliposomes and polymeric micelles—the targeting efficiency, bioavailability, and tissue accumulation capacity of sophoridine have been continuously improved. This provides a solid translational foundation for sophoridine to alleviate organ injury and improve prognosis via multi-pathway synergistic intervention in sepsis (Figure 1).

## 2 Scientific background and rationale

### 2.1 The central role of organ injury in sepsis pathogenesis and therapeutic challenges

Sepsis is defined as life-threatening organ dysfunction caused by a dysregulated host response to infection (Matot and Sprung, 2001). Its pathogenesis involves abnormal activation of the host immune system by invading pathogens, triggering uncontrolled systemic inflammation and multiple organ dysfunction, which may further progress to multiple organ failure (MOF) (Gullo, 1999). This cascade initiates when pathogen-associated molecular patterns (PAMPs) and damage-associated molecular patterns (DAMPs) released from injured cells are recognized by innate immune receptors (e.g., Toll-like receptors), activating signaling pathways such as NF-κB and Mitogen-Activated Protein Kinase (MAPK). These pathways induce the release of large quantities of pro-inflammatory cytokines (e.g., TNF-α, IL-6, IL-1β), forming a “cytokine storm” (Chousterman et al., 2017). This inflammatory cascade disseminates via the bloodstream, disrupts homeostasis, and extensively affects organs such as the heart, lungs, kidneys, and liver, constituting a primary cause of patient mortality (Carcillo and Shakoory, 2024).

Although early goal-directed therapy (EGDT), centered on fluid resuscitation and timely antibiotic administration, has significantly improved survival rates, these supportive interventions fail to fundamentally halt inflammation-mediated tissue damage as they do not target the underlying pathological mechanisms (Coba et al., 2011; Romero and Hernández, 2013). Moreover, traditional anti-inflammatory strategies often focus on single targets, whereas the pathological mechanisms of sepsis are highly heterogeneous and temporally dynamic. Single interventions are prone to compensatory pathway activation or therapeutic escape, limiting long-term efficacy. Thus, there is an urgent need to develop systemic therapeutic strategies capable of simultaneously intervening in multiple key processes.

### 2.2 Drug repositioning: an efficient strategy for complex diseases

Drug repositioning refers to exploring the therapeutic potential of approved drugs or compounds with established safety profiles for indications outside their original scope (Jourdan et al., 2020). This strategy leverages existing pharmacokinetic, toxicological, and clinical safety data, bypassing early screening and toxicity assessment stages to directly enter validation studies for new indications, thereby significantly enhancing drug development efficiency and success rates, particularly for diseases with complex mechanisms and urgent clinical needs (Liu et al., 2013; Hua et al., 2022).

**FIGURE 2:**
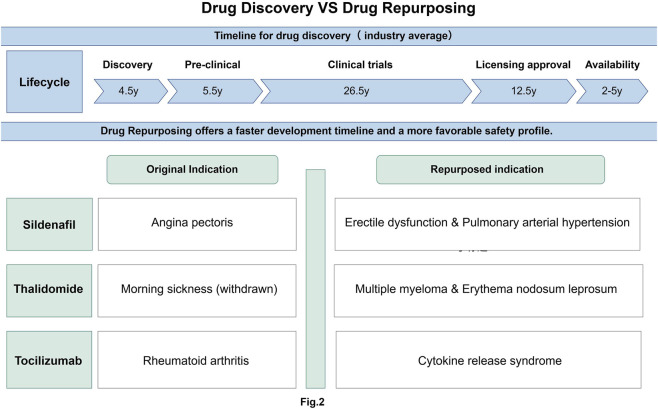
Drug repositioning strategies for complex diseases. This schematic illustrates the process and advantages of repurposing established pharmacological agents for novel clinical indications, bypassing early-stage toxicological screening to accelerate translational application in sepsis.

Numerous successful cases attest to the value of this strategy. Sildenafil was repurposed from an anti-anginal agent to an effective treatment for erectile dysfunction and pulmonary arterial hypertension (Keats et al., 2018); thalidomide transformed from a teratogen into a key therapeutic for multiple myeloma (Amare et al., 2021); tocilizumab, an IL-6 receptor antagonist, was approved not only for rheumatoid arthritis but also for severe infection-related cytokine release syndrome (Tanaka et al., 2018; Choy et al., 2020). These examples demonstrate that many “old drugs” possess multi-pathway regulatory capabilities and clinical versatility beyond their original design (Figure 2).

In the context of sepsis treatment, several existing drugs have shown multi-target potential. For instance, pentoxifylline can alleviate septic inflammation and improve microcirculation by inhibiting the NF-κB pathway (Biswas et al., 1993), and dexamethasone has been shown to reduce mortality in severe viral infection-induced sepsis-like inflammation (Kino et al., 2021). These successes provide important insights for exploring natural products with broad regulatory capabilities for sepsis treatment.

### 2.3 The multifaceted pharmacological profile of sophoridine: from existing evidence to new indication inference

Sophoridine is a natural quinolizidine alkaloid derived from *Sophora flavescens* Aiton (Fabaceae) (Li et al., 2020). Pharmacokinetic studies have demonstrated that sophoridine follows a two-compartment model in animal models, with an absolute bioavailability of approximately 41.8% (Wang et al., 2022). The tissue distribution profile of sophoridine has also been characterized in rats. Following intravenous administration of 20 mg/kg sophoridine via the tail vein, the elimination half-life and time to peak concentration (Tmax) were 1.75 ± 0.66 h and 1.97 ± 0.63 h, respectively. The area under the plasma concentration–time curve from zero to infinity $\left({\text{AUC}}_{0‐}\infty \right)$ and peak plasma concentration (Cmax) were 10.80 ± 2.69 mg mL^-1^ h and 5.56 ± 0.78 mg mL^-1^, respectively. Urinary excretion data within 24 h indicated that approximately 93% of the administered dose was excreted in urine, confirming that the kidney is the primary organ for sophoridine elimination.

Comparison of AUC values across plasma and tissues further revealed that sophoridine accumulates at relatively high levels in multiple sepsis-affected organs, including the kidney and gastrointestinal tract (Wang et al., 2022). Collectively, these findings demonstrate that sophoridine exhibits favorable pharmacological properties characterized by high tissue accumulation and a relatively long half-life (Wang et al., 2010). Notably, sophoridine exerts prominent multi-dimensional and multi-level regulatory effects, including anti-inflammatory, antioxidant, immunomodulatory, and protective activities on multiple vital organ systems.

Mechanistic studies have shown that sophoridine possesses moderate lipophilicity and efficient cell membrane permeability, enabling it to passively diffuse into cells while binding to corresponding receptors, thereby precisely modulating intracellular signaling molecules (Li et al., 2021a). These characteristics, together with its favorable bioavailability and tissue distribution, form the mechanistic basis for its systemic therapeutic effects.

Notably, recent preclinical studies have preliminarily characterized the pharmacokinetic behavior of sophoridine in septic rat models: compared with normal rats, septic rats showed a prolonged elimination half-life (2.12 ± 0.58 h), increased ${\text{AUC}}_{0‐}\infty$ (14.32 ± 3.15 mg mL^-1^ h), and higher accumulation in inflammatory injured organs (lung, liver, kidney, intestine), which may be related to the increased vascular permeability in sepsis (Chen et al., 2023). A preliminary PK-PD model has been established, which shows that the plasma concentration of sophoridine is significantly positively correlated with its inhibitory effect on TNF-α and IL-6 levels, and the effective concentration for 50% maximal effect (EC_50_) for anti-inflammatory activity is 1.28 ± 0.32 μg/mL. Nevertheless, critical knowledge gaps remain: the PK-PD model linking tissue drug concentration to organ-protective effects, and the pharmacokinetic characteristics in different stages of sepsis, have not been fully established, which are key directions for subsequent research.

Regarding anti-inflammatory activity, sophoridine significantly downregulates the expression of pro-inflammatory cytokines such as TNF-α, IL-6, and IL-1β by inhibiting the NF-κB pathway, effectively curbing excessive inflammatory cascades. This benefit has been confirmed in acute lung injury models, where it alleviates pulmonary inflammatory infiltration, protects alveolar structure, and improves gas exchange function, suggesting its potential application in respiratory diseases (Liang et al., 2022). Concerning antioxidant effects, sophoridine activates the Nuclear Factor Erythroid 2-Related Factor 2 (Nrf2)/Heme Oxygenase-1 (HO-1) signaling axis, upregulating the expression of antioxidant enzymes like HO-1, Superoxide Dismutase (SOD), and Glutathione Peroxidase (GSH-Px), efficiently scavenging reactive oxygen species, and restoring redox balance, offering significant protection under high oxidative stress conditions such as sepsis (Huang, 2020). Particularly noteworthy is its “bidirectional immunomodulatory” capacity: it inhibits the overactivation of the innate immune system, preventing the “cytokine storm,” while also promoting the reconstruction and recovery of adaptive immune function. Specifically, sophoridine can inhibit Classical Activated Macrophages (M1) macrophage polarization (preventing the cytokine storm) while promoting Alternatively Activated Macrophages (M2) macrophage activity (aiding tissue repair); it also modulates the Cluster of Differentiation 4 Positive (CD4^+^)/Cluster of Differentiation 8 Positive (CD8^+^) T cell ratio, corrects Treg cell abnormalities, and promotes the reestablishment of immune homeostasis, demonstrating multi-target and intelligent immunoregulatory advantages (Liang et al., 2022).

In septic animal models, studies on sophoridine further confirm the practical effects of these mechanistic insights. It significantly alleviates systemic inflammatory response, markedly reduces serum inflammatory cytokine concentrations, decreases abnormal lymphocyte apoptosis, and increases the expression of monocyte human leukocyte antigen-DR (mHLA-DR) on peripheral blood monocytes—the latter being an important indicator for assessing immune function recovery, further supporting its systemic immunomodulatory role (Winkler et al., 2017; Zhuang et al., 2017).

## 3 Precise alignment of Sophoridine’s targets with the pathology of sepsis-induced organ injury

### 3.1 Suppressing the cytokine storm: synergistic inhibition of NF-κB and NLRP3 pathways

Sepsis is a systemic inflammatory response syndrome (SIRS) triggered by uncontrolled local infection. Its essence lies in the host immune system’s excessive response to pathogens, leading to a “cytokine storm” that causes multiple organ dysfunction and can be life-threatening (You, 2025). Among the molecular mechanisms regulating inflammation, the NF-κB signaling pathway and NLRP3 inflammasome are recognized as two core regulatory hubs driving sepsis progression (Yue et al., 2017). They function both independently and synergistically to form a self-amplifying positive feedback loop, further amplifying systemic inflammatory responses (Zhou et al., 2020).

Specifically, the NF-κB signaling pathway serves as the central “molecular switch” regulating innate immune responses, playing a pivotal role in responding to infection and tissue damage. Its activation is primarily triggered by PAMPs (e.g., lipopolysaccharide, LPS) or DAMPs (e.g., high mobility group box 1 protein, HMGB1, or ATP) binding to pattern recognition receptors (PRRs) on the cell surface, particularly Toll-like receptor 4 (TLR4), rapidly initiating downstream signaling cascades. Taking the LPS-TLR4 signal as an example, LPS first binds to LPS-binding protein (LBP) and is transferred to Cluster of Differentiation 14 (CD14), subsequently activating the TLR4/MD2 complex, inducing its conformational change and dimerization.

**FIGURE 3:**
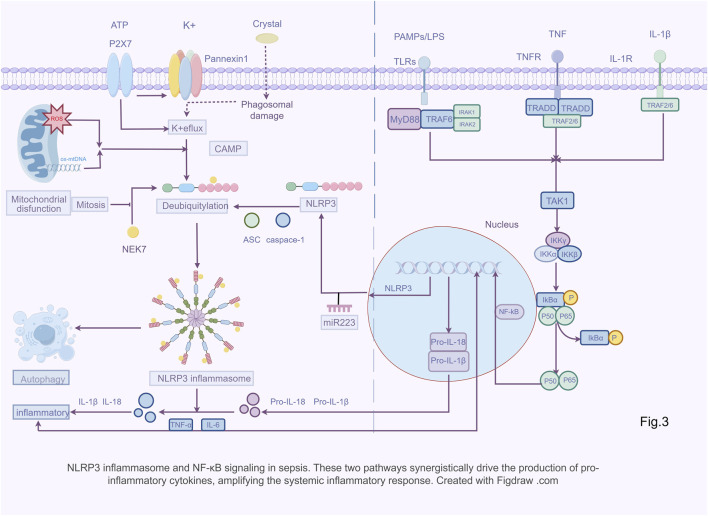
Synergistic inhibition of NF-κB signaling and NLRP3 inflammasome pathways by sophoridine. Sophoridine blocks the upstream “priming” signal via the TLR4/NF-κB axis and suppresses the downstream “activation” signal induced by mitochondrial dysfunction and ROS, effectively attenuating the systemic cytokine storm and preventing GSDMD-mediated pyroptosis. Pointed arrows represent signal transduction or molecular transitions, whereas T-bars indicate pharmacological or endogenous inhibition. Abbreviations: TLR4, Toll-like receptor 4; MyD88, myeloid differentiation primary response 88; ASC, apoptosis-associated speck-like protein containing a CARD.

This activation primarily relies on the Myeloid Differentiation Primary Response 88 (MyD88) -dependent pathway: the TLR4 intracellular TIR domain recruits MyD88, which in turn recruits Interleukin-1 Receptor-Associated Kinase (IRAK) family proteins, forming the Myddosome complex. Activated IRAK interacts with TNF Receptor-Associated Factor 6 (TRAF6), activating its E3 ubiquitin ligase activity, mediating K63-linked polyubiquitination of the Transforming Growth Factor-β-Activated Kinase 1 (TAK1) complex, thereby activating TAK1. Activated TAK1 phosphorylates the IκB Kinase β (IKKβ) subunit within the IκB Kinase (IKK) complex (comprising IKKα, IKKβ, and NF-κB Essential Modulator), prompting IKK to specifically phosphorylate Ser32 and Ser36 residues of Inhibitor of κBα (IκBα). Phosphorylated IκBα is recognized by SCF^β-TrCP, undergoes K48-linked polyubiquitination, and is degraded by the 26S proteasome. This process releases the classic NF-κB dimer (p65/p50), which is otherwise sequestered by IκBα in the cytoplasm. The free p65/p50 complex translocates into the nucleus via its nuclear localization signal (NLS), binds to κB sites (5′-GGGRNYYYCC-3′) in the promoter regions of target genes, and recruits transcriptional coactivators like CREB-Binding Protein/p300 (CBP/p300), the Mediator complex, and the basal transcriptional machinery through the transactivation domain (TAD) of p65, significantly enhancing the transcription of pro-inflammatory genes (e.g., TNF-α, IL-6, IL-1β) (Oeckinghaus and Ghosh, 2009; Hu et al., 2020; Li et al., 2021b). For instance, in septic models, LPS-induced NF-κB activation can increase TNF-α mRNA levels within 30 min, peaking at 2–4 h, highlighting the pathway’s rapid response capability in inflammation (Figure 3).

Concurrently, the NLRP3 inflammasome acts as a key “executor” in innate immune responses, and its activation strictly depends on the classical “two-signal model.” The first signal, provided by PAMPs or DAMPs via PRRs like TLRs, activates the NF-κB pathway, upregulating NLRP3 gene transcription and promoting the accumulation of precursor IL-1β (pro-IL-1β) and pro-IL-18, completing the “priming” stage of the inflammasome (Hu et al., 2024; Du et al., 2023). This process not only provides the protein foundation for subsequent activation but may also regulate NLRP3 activity through post-translational modifications (e.g., deubiquitination or phosphorylation). The second signal originates from various intracellular stresses, such as mitochondrial dysfunction-induced overproduction of mitochondrial ROS (mitoROS), potassium efflux, lysosomal rupture releasing cathepsins, or phagosome damage releasing stimulants, as well as ATP-induced ion imbalance via P2X Purinoceptor 7 (P2X7) receptor activation. These stimuli prompt NLRP3 conformational changes, leading to the recruitment of the adapter protein Apoptosis-Associated Speck-Like Protein Containing a CARD (ASC) via Pyrin Domain (PYD) domain interactions, and subsequently procaspase-1 via Caspase Recruitment Domain (CARD-CARD) interactions, assembling the functional NLRP3 inflammasome complex. The complex facilitates the autoproteolytic activation of caspase-1, which then cleaves pro-IL-1β and pro-IL-18 into mature pro-inflammatory cytokines and also cleaves Gasdermin D (GSDMD), releasing its N-terminal pore-forming domain. The latter oligomerizes to form pores in the cell membrane, causing osmotic imbalance, cell swelling, rupture, and triggering pyroptosis. This process is accompanied by the release of IL-1α, High Mobility Group Box 1 Protein (HMGB1), and other DAMPs, further activating neighboring immune cells and forming a positive feedback loop that amplifies local or systemic inflammation, playing a key driving role in chronic inflammatory diseases like atherosclerosis, type 2 diabetes, and neurodegenerative disorders (Su et al., 2018; Giamarellos-Bourboulis et al., 2011).

Notably, NF-κB and NLRP3 form a synergistically amplified positive regulatory circuit: NF-κB not only primes NLRP3 expression but also provides the necessary preconditions for its activation; conversely, NLRP3-mediated IL-1β release can enhance NF-κB activity, creating a vicious cycle of inflammation begetting more inflammation (Jiao et al., 2021; Zhang et al., 2022). This interplay makes the inflammatory response difficult to control once initiated, becoming a significant driver of sepsis deterioration.

Sophoridine’s multi-target nature enables it to synergistically intervene in this core NF-κB/NLRP3 inflammatory circuit. Its mode of action involves dual upstream and downstream blockade: it inhibits the NF-κB signaling pathway upstream, reducing cytokine production and weakening the priming signal for the NLRP3 inflammasome; downstream, the insufficient priming signal indirectly inhibits NLRP3 assembly/activation and subsequent pyroptosis. This synergistic intervention more effectively contains the inflammatory cascade amplification, demonstrating systemic anti-inflammatory advantages (Huang et al., 2011). Of particular importance, sophoridine exhibits a differential molecular regulatory profile in heterogeneous cell populations. Its action intensity, target preference, and functional output are not uniform; instead, they are highly dependent on the cell type–specific signaling microenvironment, receptor expression profile, and basal inflammatory status. In macrophages, sophoridine predominantly downregulates the transcription of *Tnf-α*, *Il-6*, and *Nlrp3* by suppressing the TLR4–MyD88–NF-κB axis, thereby dually attenuating both the priming and activation steps of inflammatory response (Zhuang et al., 2020). In parenchymal epithelial cells of the lung and kidney, it preferentially blocks NF-κB nuclear translocation and the expression of downstream pyroptotic executioners such as GSDMD-N, consequently alleviating epithelial barrier disruption and transcellular inflammatory leakage (Huang et al., 2011). Furthermore, in immune effector cells including T lymphocytes and neutrophils, sophoridine moderately modulates NF-κB-dependent survival signaling and non-canonical NLRP3 activation pathways (e.g., the caspase-11/4–GSDMD axis), further expanding its anti-inflammatory spectrum (Zhuang et al., 2020). Such hierarchical, cell-specific differential regulation enables sophoridine to not only precisely restrain excessive activation of immune cells but also concurrently protect organ parenchymal cells from pyroptosis and functional failure (Su et al., 2024). As a result, sophoridine achieves spatially compartmentalized intervention of inflammation and coordinated restoration of tissue homeostasis.

### 3.2 Maintaining organ barrier integrity: dual mechanisms of anti-apoptosis and promotion of repair

During sepsis progression, excessive apoptosis is a key mechanism leading to multiple organ dysfunction syndrome (MODS) (Lelubre and Vincent, 2018).

Research shows that while sophoridine selectively induces apoptosis in cancer cells (Peng et al., 2020), it exerts significant protective effects on normal tissue cells by inhibiting aberrant programmed cell death. At the molecular level, sophoridine’s anti-apoptotic effect is primarily achieved by upregulating anti-apoptotic members like B-Cell Lymphoma 2 (Bcl-2) and B-Cell Lymphoma-extra Large (Bcl-xL), while inhibiting the activation and translocation of pro-apoptotic proteins such as Bcl-2-Associated X Protein (Bax) and Bcl-2 Antagonist/Killer (Bak). This bidirectional regulation helps maintain mitochondrial outer membrane stability, preventing the collapse of mitochondrial membrane potential and thereby blocking the abnormal release of cytochrome c from mitochondria into the cytoplasm. Cytochrome c release is the “trigger” for activating the downstream caspase cascad (Wang et al., 2015; Dai et al., 2022). Through this mechanism, sophoridine effectively curbs the initiation of the classic intrinsic apoptotic pathway, protecting parenchymal cells from programmed death.

Furthermore, sophoridine’s protective effect also actively participates in the repair and reconstruction of critical organ barrier functions. Existing *in vitro* studies have confirmed that sophoridine can upregulate the expression of tight junction proteins (occludin, claudin-1, ZO-1) in intestinal epithelial cells, increase transepithelial electrical resistance (TEER), and reduce monolayer permeability, thereby directly protecting intestinal epithelial barrier integrity (Xie et al., 2026). This barrier-protective effect is further verified in septic animal models, where sophoridine inhibits endotoxin translocation from the intestinal tract into the blood circulation, blocks the “gut-lung axis” and “gut-liver axis” inflammatory amplification loops, and reduces secondary organ injury. Notably, the barrier-protective effect of sophoridine is not limited to the intestinal tract; it has also been shown to stabilize the vascular endothelial barrier and alveolar epithelial barrier in sepsis, showing a pan-barrier protective effect across multiple organs.

### 3.3 Reestablishing immune homeostasis: bidirectional regulation from immune activation to suppression

The immunopathological process of sepsis is complex and dynamic, characterized by a “biphasic imbalance” (Yadav and Cartin-Ceba, 2016), primarily manifesting as intense hyperinflammation in the early stage and a widespread immunosuppressive state later in the disease course.

Studies have demonstrated that, tailored to the pathological characteristics of sepsis, sophoridine possesses a unique advantage: it exerts differential regulation in a disease stage-dependent manner, rather than simply suppressing or activating the immune system (Liu et al., 2024). During the peak inflammatory phase, sophoridine acts to reduce the release of inflammatory cytokines. Upon entering the immunosuppressive phase in late sepsis, it switches its role to that of an immune awakener (Zhou et al., 2010). At this stage, sophoridine promotes the maturation of dendritic cells (DCs) and enhances the expression of MHC-II molecules on their surface, thereby restoring impaired antigen-presenting function and laying the foundation for initiating specific immune responses.

In septic animal models, sophoridine has been shown to directly upregulate IFN-γ secretion in CD4^+^ T cells, reverse T-cell exhaustion, and restore Th1-type cellular immune response (Zhao et al., 2021a). Meanwhile, it can inhibit the abnormal proliferation of immunosuppressive regulatory T cells (Tregs), downregulate the expression of immune checkpoint molecules (PD-1, TIM-3) on exhausted T cells, break the immune-tolerant microenvironment in late sepsis, and enhance the host’s anti-infective defense capability. These findings directly confirm the bidirectional immunomodulatory effect of sophoridine, rather than speculative inference from structural analogues.

The above-proposed bidirectional regulatory hypothesis of sophoridine—switching from anti-inflammation to immune restoration—is based on its multiple pharmacological activities observed in diverse *in vitro* models. Nevertheless, the clinical translation of this hypothesis faces a critical challenge: the realization of its biphasic action is highly dependent on precise dosage and therapeutic time window. Current studies have mostly focused on acute inflammation models (e.g., administration 2–6 h after LPS challenge at doses of 2.5–9 mg/kg) (Liang et al., 2022), confirming its protective effects during the cytokine storm phase. However, the optimal dosage and delayed administration timing required for its immunostimulatory effects have not been systematically evaluated in models of the immunosuppressive phase of sepsis.

Therefore, future studies are urgently needed to design controlled experiments in animal models, clarifying the distinct or even opposing effects on immune indices when administered at different stages: the early phase (0–24 h) versus the late phase (>24–48 h) of the disease. This will provide precise pharmacological evidence for developing sophoridine into a time-sequenced immunotherapeutic agent. The key to future investigation lies in accurately determining the intervention timing and dosage—enabling a transition from anti-inflammation to immune activation based on the immune dynamics of sepsis—to avoid associated risks.

## 4 Horizontal comparison of the efficacy of repurposed drugs in organ protection among patients with sepsis

### 4.1 Comparative analysis of various repurposed drugs for sepsis treatment

Among the currently available anti-inflammatory and immunomodulatory agents on the market, many exhibit potential at specific mechanistic levels, yet their scope of action or clinical applicability remains limited (Kanashiro et al., 2017). Following a comprehensive literature review, we performed a comparative analysis of the transverse therapeutic efficacy of the following four drugs in the field of drug repurposing for sepsis, using the regulation of the core sepsis pathways—NF-κB and NLRP3 inflammasome—as the key reference criterion.

**FIGURE 4:**
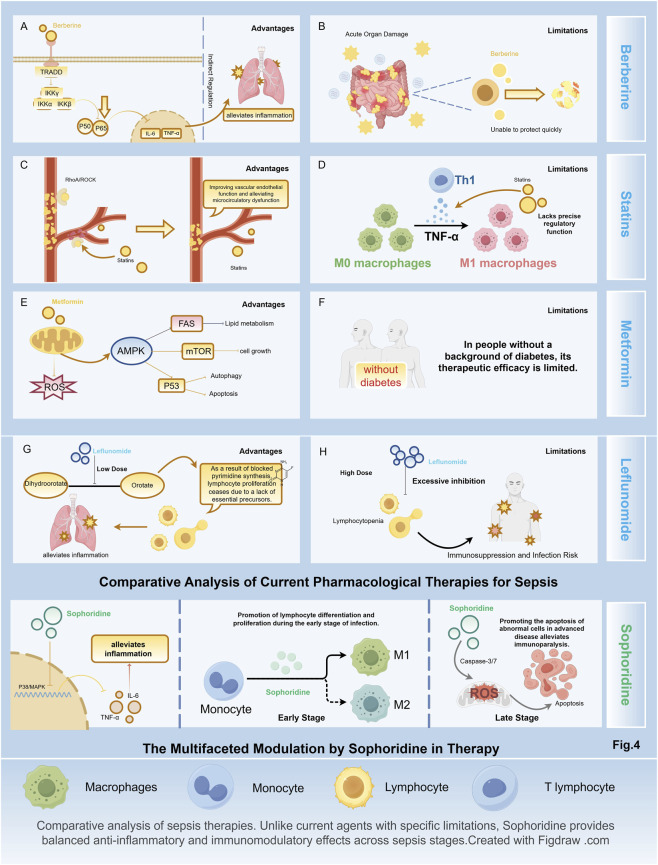
Comparative pharmacological profiles of repurposed agents versus stage-specific immunomodulation by sophoridine in sepsis. **(A)** Anti-inflammatory signaling cascade and therapeutic merits of berberine; **(B)** Limitation of delayed organ-protective potency for berberine; **(C)** Vascular endothelial protection and mechanistic benefits of statins via RhoA/ROCK signaling; **(D)** Deficient precise tuning of macrophage polarization as a key limitation of statins; **(E)** AMPK-governed metabolic, autophagic, and apoptotic regulatory advantages of metformin; **(F)** Attenuated therapeutic efficacy of metformin in non-diabetic individuals; **(G)** Low-dose leflunomide mitigates pulmonary inflammation through blockade of pyrimidine synthesis and excess lymphocyte proliferation; **(H)** High-dose leflunomide triggers overt immunosuppression, lymphocytopenia, and elevated infection susceptibility. The bottom panel depicts bidirectional regulation by sophoridine: it facilitates monocyte differentiation during early.

**FIGURE 5:**
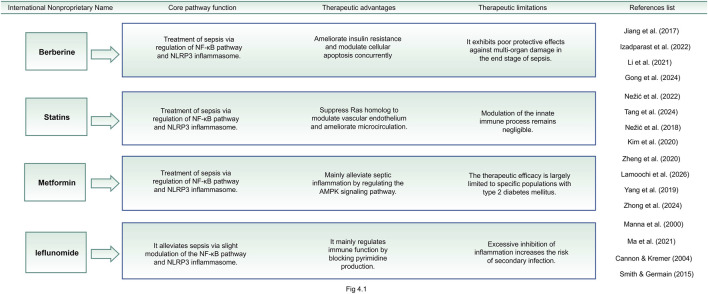
Core pathway functions and clinical limitations of selected repurposed drugs for sepsis. Detailed comparison of berberine, statins, metformin, and leflunomide regarding their regulatory capacity on NF-κB/NLRP3 pathways, organ-specific protective effects, and inherent secondary side effects. The table summarizes the evidence-based advantages and limitations for each agent to highlight the comparative superiority of network-regulating alkaloids.

To ensure analytical objectivity, we evaluated these repurposed agents across three consistent dimensions: primary anti-inflammatory mechanism, direct parenchymal organ protection, and clinical limitations in septic settings. For instance, while berberine effectively inhibits the NF-κB and NLRP3 pathways to alleviate systemic inflammation and insulin resistance, it exhibits weak direct protective effects against multi-organ damage in the end stage of sepsis (Li et al., 2024), rendering it insufficient for terminal multiple organ dysfunction (Jiang et al., 2017; Li et al., 2021c; Izadparast et al., 2022; Gong et al., 2024). Statins can ameliorate microcirculation by suppressing Rho homolog to modulate the vascular endothelium; however, their direct regulatory capacity on core innate immune processes remains negligible, and myotoxicity risks limit their intensive care utility (Nežić et al., 2018; Kim et al., 2020; Nežić et al., 2022; Tang et al., 2024). Metformin mitigates septic inflammation primarily by regulating the AMPK signaling pathway, yet its therapeutic efficacy is largely restricted to specific populations with concurrent type 2 diabetes mellitus (Yang et al., 2019; Zheng et al., 2020; Zhong et al., 2024; Lamoochi et al., 2026). Lastly, leflunomide suppresses excessive inflammation by blocking pyrimidine production, but this non-selective inhibition inherently compromises the host’s pathogen-clearing capability, significantly increasing the risk of secondary infections (Manna et al., 2000; Cannon and Kremer, 2004; Smith and Germain, 2015; Ma et al., 2021; Fragoso and Brooks, 2015) (Figures 4, 5).

In direct contrast to the above-mentioned repurposed drugs, sophoridine exhibits more comprehensive and sepsis-specific multi-target regulatory capabilities. Existing preclinical studies have confirmed that sophoridine can inhibit p38 MAPK phosphorylation, block the release of pro-inflammatory cytokines, and simultaneously modulate apoptotic pathways to protect parenchymal cells from sepsis-induced injury (Zhao et al., 2021a). In septic mouse models, sophoridine significantly improves the 7-day survival rate, reduces serum levels of TNF-α, IL-6 and IL-1β, and alleviates pathological injury in the lung, liver, kidney and intestine, showing direct multi-organ protective effects (Xie et al., 2026). Notably, sophoridine exhibits a unique bidirectional immunoregulatory profile that matches the biphasic progression of sepsis: it suppresses the cytokine storm in the early hyperinflammatory phase, while reversing immune exhaustion and enhancing anti-infective immunity in the late immunosuppressive phase (Liang et al., 2022). These findings fully demonstrate that sophoridine has a bidirectional immunomodulatory profile that matches the biphasic pathological progression of sepsis. Notably, the specific molecular mechanism of its stage-dependent differential regulation and the optimal intervention window in late sepsis still need to be further verified in standardized biphasic sepsis animal models.

As a quinolizidine alkaloid, our previous study revealed that sophoridine reduces infarct volume and alleviates cerebral edema in a rat model of focal cerebral ischemia by downregulating TRAF6 expression and upregulating ERK1/2 phosphorylation in the ischemic cerebral cortex. Since these pharmacological effects rely on effective drug penetration into the brain parenchyma and action on target cells, these findings suggest that sophoridine may possess favorable blood–brain barrier (BBB) permeability. Furthermore, structurally related Sophora alkaloids, such as matrine and oxymatrine, have been validated to efficiently cross the BBB, providing theoretical support for the BBB-penetrating potential of sophoridine. Recent *in vivo* studies have directly verified the BBB penetration capacity of sophoridine in SAE mouse models: after intravenous administration of 20 mg/kg sophoridine, the drug can be detected in brain tissue within 30 min, with a brain/plasma concentration ratio of 0.32 ± 0.08 at 2 h after administration (Xie et al., 2026). Immunofluorescence staining further confirmed that sophoridine can upregulate the expression of tight junction proteins ZO-1 and Occludin in brain microvascular endothelial cells, reduce BBB permeability, and inhibit the infiltration of peripheral inflammatory cells into the brain parenchyma. Meanwhile, pharmacodynamic studies showed that sophoridine can downregulate microglial overactivation, inhibit the release of inflammatory factors in the hippocampus, and improve cognitive dysfunction in septic mice, providing direct preclinical evidence for its application in SAE. It should be noted that the long-term safety of sophoridine in the central nervous system and its optimal dosage regimen for SAE still need to be further verified in large animal models.

### 4.2 Classification of major therapeutic paradigms for sepsis

Currently, in the treatment of sepsis, traditional “single-target” therapeutic strategies can precisely block specific inflammatory pathways. However, in the face of the complex interplay of pathological mechanisms such as immune dysregulation, oxidative stress, and cell death, these approaches are often compromised by issues such as compensatory rebound and therapeutic escape, ultimately limiting their efficacy. Contemporary treatment paradigms are shifting toward “multi-pathway synergistic regulation,” emphasizing systemic intervention and the dynamic equilibrium of biological networks (Kaur and Silakari, 2017; Makhoba et al., 2020).

Current therapeutic strategies for sepsis-related organ injury can be broadly categorized based on their core mechanisms: (1) Anti-inflammatory agents targeting the NF-κB signaling pathway: These drugs inhibit IKK kinase activity or prevent IκBα degradation to block NF-κB nuclear translocation, thereby reducing excessive release of pro-inflammatory cytokines such as TNF-α and IL-6 (Feng et al., 2023). (2) Biologic agents (e.g., anti-TNF-α antibodies).: These agents potently neutralize circulating inflammatory mediators to rapidly alleviate systemic cytokine storms, but may compromise host immune surveillance and increase the risk of secondary infections (Peluso and Palmery, 2016; Souza et al., 2023) (3) Antioxidant agents (e.g., N-acetylcysteine, NAC).: These drugs primarily scavenge excess reactive oxygen species (ROS) to mitigate mitochondrial damage and protect cellular energy metabolism. Albuszies and Brückner (2003) However, such agents generally have short *in vivo* half-lives, uneven tissue distribution, and poor targeting, leading to suboptimal clinical translation and difficulty achieving ideal organ protection (Rocha et al., 2012).

Against this backdrop, the emergence of sophoridine offers new possibilities for sepsis treatment. Studies show it can inhibit NF-κB nuclear translocation, reducing pro-inflammatory cytokine levels in organs like the lungs, liver, and kidneys (Huang et al., 2011); block NLRP3 inflammasome activation and GSDMD-mediated pyroptosis, containing the inflammatory cascade’s spread (Xie et al., 2026). Simultaneously, existing studies have confirmed that sophoridine can directly activate the PI3K/Akt/mTOR signaling pathway in epithelial cells, promote the regeneration of alveolar and intestinal epithelial cells, and accelerate the repair of organ barrier damage in sepsis (Tang et al., 2022). While sophoridine requires further direct validation in these specific regenerative pathways, its broad, overlapping regulatory profile thoroughly aligns with a “network-regulating drug” therapeutic paradigm.

## 5 From mechanism integration to paradigm innovation: deepening and prospects of Sophoridine's multi-target therapy

### 5.1 Paradigm transcendence: systemic intervention from a network pharmacology perspective

Sophoridine’s role in sepsis treatment signifies a paradigm shift from “single-target, single-effect” to “multi-target, network regulation” systemic intervention.

Network pharmacology analysis reveals that sophoridine’s action targets overlap with multiple sepsis-related disease targets. Functional analysis (GO) shows it simultaneously covers core pathways like NF-κB, NLRP3, MAPK, and PI3K/Akt, enabling concurrent intervention in the cytokine storm, cellular stress, and immune imbalance. It also participates in inflammation regulation and profoundly influences fundamental life processes like oxidative stress balance, mitochondrial metabolism, and cytoskeletal remodeling. Multi-omics data further reveal that its targets (e.g., Albumin, MAPK1) are often hub proteins that can widely affect oxidative stress, mitochondrial metabolism, etc.,; Kyoto Encyclopedia of Genes and Genomes (KEGG) pathway enrichment analysis also confirms its involvement in various key septic pathways like Toll-like receptor signaling, apoptosis, and autophagy (Shang et al., 2018).

This network-based regulation endows it with significant “biological fault tolerance”—even if one pathway is compensated or mutated, other targets can maintain overall efficacy, reducing the risk of drug resistance. Molecular docking studies indicate that sophoridine can stably bind to multiple amino acid residues of NF-κB p65 and NLRP3, forming a “multi-point anchoring” mode, structurally avoiding therapeutic escape caused by single-point mutations.

### 5.2 The other side of complexity: deep scientific questions and challenges posed by multi-target property

When dealing with highly complex systemic inflammatory syndromes like sepsis, while multi-target drugs are theoretically considered ideal strategies for simultaneously intervening in multiple pathological pathways, their “one-drug, multiple-effects” model—seemingly fitting the dynamic, multi-dimensional pathological features of sepsis—actually masks several blind spots in clinical translation:

1. Precise control of intervention timing. Sepsis is a dynamically evolving process (Delano and Ward, 2016), from the cytokine storm to immunosuppression, where timing is crucial. Early inhibition of NF-κB/MAPK pathways might control the condition, but continued anti-inflammatory action during the immune paralysis phase can worsen immunosuppression, increasing infection risk, and counterproductively affecting treatment. Unfortunately, current multi-target drug development emphasizes the number of targets but often neglects the critical aspect of “when to intervene,” reducing “precision therapy” to a mere concept. In this context, sophoridine is endowed with the high hope of “bidirectional regulation”—suppressing inflammation early and restoring immunity late—but evidence for its clinical reproducibility and stability is insufficient. The core challenge is whether its pharmacokinetics can match disease phase changes: can blood concentrations effectively inhibit inflammatory responses during the peak phase while maintaining activity to restore immune function during the immunosuppressive trough? This requires Pharmacokinetics-Pharmacodynamics (PK-PD) model support, for which existing data are extremely scarce (Lu et al., 2012).
2. Cellular heterogeneity and microenvironmental differences. Beyond the macroscopic challenge of temporal control, multi-target drugs face a deeper microscopic dilemma: they are further constrained by cellular heterogeneity and microenvironmental differences. Taking sophoridine as an example, its effects vary across different immune cell subsets (e.g., macrophages, neutrophils) and heterogeneous cellular microenvironments in sepsis, with sometimes even contradictory effects depending on the cell type and activation state. While it can promote M1-to-M2 transformation and alleviate endothelial damage *in vitro*, these findings are difficult to replicate in the complex, variable overall pathological process of sepsis. Drug efficacy is highly “context-dependent”: the same component might activate opposing pathways (Shapouri-Moghaddam et al., 2018). For instance, TRAF protein expression varies across tissues, potentially causing sophoridine to exert anti-inflammatory effects in the liver while disrupting immunity in the spleen or lungs (Ha et al., 2009). Microenvironment-driven pharmacological differentiation exposes the risks of “non-targeting” or “mis-targeting” associated with traditional administration strategies, highlighting the limitations of non-targeted approaches. Future development must shift from extensive multi-target screening to intelligent delivery systems capable of sensing the spatiotemporal microenvironment.

### 5.3 From mechanism enumeration to paradigm construction: the new precision therapy strategy for sepsis led by sophoridine

**FIGURE 6:**
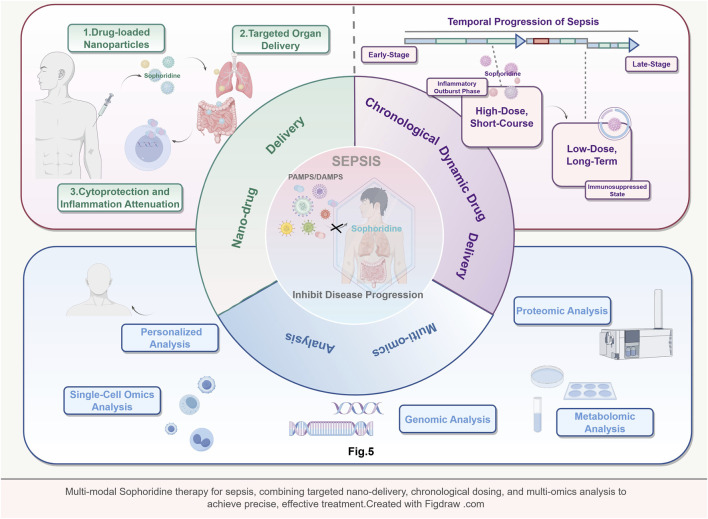
Precision therapy framework combining nanotechnology and multi-omics. A closed-loop strategy comprising: (1) intelligent nano-delivery systems (e.g., drug-loaded nanoparticles): for targeted organ delivery and spatiotemporal drug release; (2) chronological dynamic dosing (high-dose/short-course in early stages vs. low-dose/long-term in late stages) to match sepsis progression; and (3) single-cell and spatial multi-omics (genomic, proteomic, and metabolomic analyses) for decoding dynamic immune network interactions. This integrated paradigm aims to transition sophoridine treatment toward personalized medicine.

As mentioned regarding the pathogenesis of sepsis, the disease itself is a comprehensive outcome resulting from the interaction of multiple modules and pathways at different time points. Precisely due to the extreme complexity of the interaction between sepsis and sophoridine’s therapeutic effects, we can no longer be satisfied with traditional linear research paradigms (Peterson and Chase, 2017). Therefore, future treatment strategies should elevate the “multi-target” therapeutic value provided by sophoridine from a static therapeutic attribute to a dynamic, controllable strategic foundation. To address the aforementioned challenges of temporality and heterogeneity, sophoridine’s development must transcend the traditional ‘drug-disease’ binary paradigm and instead construct a new precision therapy framework. This framework consists of three mutually supporting pillars: a biomarker-driven dynamic dosing strategy (addressing “when to intervene”), a nanotechnology-enabled spatiotemporally controlled targeted delivery system (addressing “where to intervene”), and a multi-omics-guided systems pharmacology analysis (elucidating “how to intervene”). Only through the synergy of these three can sophoridine’s multi-target potential be transformed from an unpredictable property into a precisely programmable therapeutic advantage (Figure 6).

#### 5.3.1 Sepsis temporal biomarker-driven dynamic dosing strategy

Addressing sepsis requires breaking through the “temporal” bottleneck, promoting the transition of multi-target drugs from fixed dosing to dynamic intervention. The core lies in constructing an intelligent dosing system driven by biomarkers. The disease does not progress linearly but evolves from a “cytokine storm” to “immune paralysis,” requiring differential regulation at different stages: early inhibition of NF-κB, late prevention of immune exhaustion. The traditional “one-size-fits-all” model is ill-suited to this dynamic process. The key to a breakthrough is establishing a real-time immune phenotyping system based on HMGB1, mHLA-DR, and cytokine profiles, enabling precise identification of the disease stage (Saxena et al., 2024; Xu et al., 2025). Leveraging this, sophoridine’s multi-target characteristics can be elevated to a “programmable signal regulation tool.” (Zhang and Ning, 2021) Guided by biomarkers, high-dose, short-course regimens tackle the inflammatory peak, while low-dose, long-cycle regimens aid immune reconstruction (Jeon et al., 2025). Thus, treatment moves from empirical to an “algorithm-driven, on-demand response” precision adaptive model, taking a critical step towards precision medicine.

#### 5.3.2 Spatiotemporal targeted drug delivery: nanotechnology-based delivery systems

To precisely deliver sophoridine within the complex pathological microenvironment of sepsis, it is urgent to overcome the pharmacokinetic limitations of conventional systemic administration, including rapid plasma clearance, imbalanced tissue distribution, poor trans-endothelial transport, and lack of selectivity for target cell uptake (Mitchell et al., 2021; Mittal et al., 2018; Waheed et al., 2022). Although sophoridine exerts multi-target pharmacological activities (e.g., regulating the NF-κB, NLRP3, and mitochondrial apoptotic pathways), its therapeutic efficacy in the highly heterogeneous sepsis microenvironment is vulnerable to target cell type, activation status, and subcellular localization. For instance, sophoridine effectively inhibits TNF-α/IL-6 secretion from LPS-activated M1 macrophages, yet its effects on the JAK-STAT/Foxp3 axis in endothelial cells or regulatory T cells (Tregs) remain unclear, and may even disturb immune homeostasis. More critically, sepsis-induced organ injury follows a dynamic evolution: the early stage is dominated by barrier disruption and neutrophil infiltration, whereas the late phase shifts toward immunosuppression and fibrosis. Static dosing regimens can hardly adapt to such sequential pathological progression. Therefore, the future development priority lies in integrating sophoridine with smart nanocarriers to construct a targeting-responsive delivery system capable of sensing and responding to the disease microenvironment.

Herein, we propose a rational design framework. First, biocompatible materials such as poly(lactic-co-glycolic acid) (PLGA) nanoparticles or liposomes are selected to fabricate nanocarriers for sophoridine encapsulation, thereby improving its solubility and stability (Hu, 2012). Subsequently, surface functionalization is performed to conjugate targeting ligands that specifically recognize overexpressed molecules in septic lesion sites (Ur Rashid et al., 2020; Cui et al., 2025). For example, conjugation with anti-intercellular adhesion molecule-1 (ICAM-1) antibodies or vascular endothelial cadherin-targeting peptides enables active targeting of nanoparticles to activated inflammatory endothelial cells, facilitating enrichment in injured organs including the lungs and kidneys. For controlled drug release, environment-responsive materials are engineered to exploit pathological signals unique to lesion sites, such as acidic pH, high reactive oxygen species (ROS) levels, or overexpressed matrix metalloproteinase-9 (MMP-9). Specifically, thioketal linkages or ROS-sensitive phenylboronate ester bonds are employed as connecting bridges or carrier backbones, ensuring that the drug remains inert before reaching target sites and is only triggered for on-demand release in inflammatory regions with high ROS levels.

This spatiotemporal synergistic strategy combining active targeting and triggered release aims to achieve two major objectives: on the one hand, it markedly elevates the local concentration of sophoridine in diseased tissues, thereby enhancing its regulatory potency on key pathways including inflammation and cell death; on the other hand, it minimizes drug distribution in non-target tissues (e.g., healthy organs), fundamentally reducing potential systemic toxicity and off-target risks. Nevertheless, the *in vivo* application of nanocarriers faces severe challenges; in particular, rapid recognition and clearance by the mononuclear phagocyte system (MPS) following intravenous administration drastically compromises delivery efficiency (Lu et al., 2023).

Through this strategy, sophoridine is expected to transform from a broad-spectrum natural molecule into a spatiotemporally controllable and programmable therapeutic tool. To transition this system from a conceptual model to an actionable technical route, a rigorous three-step verification scheme is required: (1) Formulation optimization: evaluating encapsulation efficiency and triggered-release kinetics in simulated acidic/ROS-rich septic plasma; (2) Biodistribution tracking: utilizing near-infrared fluorescence (NIRF) imaging to monitor spatiotemporal accumulation in specific injured organs; and (3) Therapeutic validation: comparing the targeted nanocarrier against free sophoridine in cecal ligation and puncture (CLP) models using precise PK/PD endpoints. This closed-loop experimental design addresses the core bottlenecks of “inaccurate targeting” and “temporal mismatch,” establishing a concrete pathway toward clinical translation.

#### 5.3.3 Multi-omics guided network analysis of disease mechanisms

Current mechanistic studies of sepsis predominantly focus on isolated molecular events such as NF-κB signaling activation or mitochondria-dependent apoptosis (Liu and Liang, 2025; Tong et al., 2025; Lin et al., 2025; Zhao et al., 2021b). Such reductionist approaches fail to systematically resolve the core pathological feature of sepsis: cross-organ inflammatory network disturbance driven by imbalanced dynamic crosstalk among multiple cell types. This limitation leaves the field trapped in a “mechanistic black box” and incapable of decoding the global pathological landscape of the disease. As a natural alkaloid with well-defined multi-target pharmacological activities, sophoridine exerts nonlinear *in vivo* effects that reflect systemic reprogramming of the septic pathological microenvironment, rather than mere inhibition of a single pathway. Therefore, it is imperative to transcend reductionist paradigms and establish a systems pharmacology framework integrated with single-cell multi-omics to achieve a paradigm shift from “empirical intervention” to “panoramic mechanistic decoding”.

Specifically, in nanocarrier-mediated sepsis intervention models, the following strategies should be applied synergistically: single-cell RNA sequencing (scRNA-seq) to delineate heterogeneous response landscapes across immune cells, endothelial cells, and parenchymal cells (e.g., renal proximal tubular epithelial cells, alveolar type I/II epithelial cells); and spatial transcriptomics to define the spatial distribution of critical cell populations and their neighborhood interaction patterns (Dai et al., 2024). This integrated strategy addresses three urgently unresolved key scientific questions: (1) Do the therapeutic cellular targets of sophoridine exhibit tissue specificity, and does sophoridine primarily act on specific subsets of the myeloid or lymphoid lineage? (2) Does sophoridine regulate macrophage function by promoting phenotypic polarization from M1-like to M2-like macrophages, or via epigenetic reprogramming through modulation of the STAT6/PPARγ axis? (3) Is there functional coupling among the responses of different cell types during sophoridine treatment (Qiao and Cui, 2022; Huang et al., 2025)?

Notably, these multi-dimensional omics datasets constitute a self-validating, positively feedback closed-loop analytical system. If scRNA-seq and RNA velocity-based trajectory inference confirm that therapeutic efficacy strongly depends on functional enhancement of Treg subsets, the findings can directly guide the design of targeting ligands on nanocarriers to achieve cell-subset-specific delivery (Liu and Liang, 2025). Thus, “multi-omics-driven mechanistic network modeling” serves not only as the core engine for decoding the efficacy code of sophoridine, but also as the critical hub bridging precise nano-delivery and personalized immune intervention. Ultimately, this approach promotes the establishment of a closed-loop translational medicine system characterized by clear mechanisms, precise targeting, controllable efficacy, and feedback optimization.

## 6 Conclusion

The repurposing of sophoridine for sepsis treatment provides a preclinical theoretical basis for the transition from traditional single-target therapy to multi-pathway network regulatory strategy for complex diseases. The precision therapeutic framework proposed herein—integrating biomarker-driven dynamic dosing, nanotechnology-enabled targeted delivery, and multi-omics-guided mechanistic decoding—offers a referable research approach for the preclinical development of sophoridine in sepsis treatment. However, the clinical translation of this framework is severely constrained by critical limitations. First, target redundancy within sepsis immune networks implies that sophoridine’s intervention could be bypassed by compensatory inflammatory cascades. Second, the profound temporal-spatial heterogeneity of sepsis makes a universal dosing regimen unfeasible; the exact therapeutic window for transitioning from immune suppression to stimulation remains theoretically derived. Third, significant individual variations in basal immunity and pharmacokinetics limit the predictability of clinical efficacy. Consequently, the application of this framework must be strictly confined to controlled preclinical models. Future research must prioritize definitive pharmacokinetic/pharmacodynamic (PK/PD) profiling and rigorously address these mechanistic boundaries before any clinical implementation can be considered.

## Glossary

AMPK: Adenosine Monophosphate-Activated Protein Kinase
ASC: Apoptosis-Associated Speck-Like Protein Containing a CARD
Akt (PKB): Protein Kinase B
Bax: Bcl-2-Associated X Protein
Bak: Bcl-2 Antagonist/Killer
Bcl-2: B-Cell Lymphoma 2
Bcl-xL: B-Cell Lymphoma-extra Large
CARD: Caspase Recruitment Domain
CBP/p300: CREB-Binding Protein/p300
Caco-2: Colon Adenocarcinoma Cell Line
CD4+: Cluster of Differentiation 4 Positive
CD8+: Cluster of Differentiation 8 Positive
CD14: Cluster of Differentiation 14
DAMPs: Damage-Associated Molecular Patterns
DCs: Dendritic Cells
EGDT: Early Goal-Directed Therapy
GSDMD: Gasdermin D
GO: Gene Ontology
GSH-Px: Glutathione Peroxidase
HMGB1: High Mobility Group Box 1 Protein
HO-1: Heme Oxygenase-1
IKK: IκB Kinase
IκBα: Inhibitor of κBα
IKKα: IκB Kinase α
IKKβ: IκB Kinase β
IL: Interleukin
IL-1β: Interleukin-1β
IL-6: Interleukin-6
IL-10: Interleukin-10
IL-18: Interleukin-18
IRAK: Interleukin-1 Receptor-Associated Kinase
IRB: Institutional Review Board
KEGG: Kyoto Encyclopedia of Genes and Genomes
LBP: LPS-Binding Protein
LPS: Lipopolysaccharide
MAPK: Mitogen-Activated Protein Kinase
mHLA-DR: Monocyte Human Leukocyte Antigen-DR
M1: Classical Activated Macrophages
M2: Alternatively Activated Macrophages
mTOR: Mammalian Target of Rapamycin
MODS: Multiple Organ Dysfunction Syndrome
MyD88: Myeloid Differentiation Primary Response 88
NAC: N-Acetylcysteine
NF-κB: Nuclear Factor-κB
NF-κB p65/p50: NF-κB Subunits p65 and p50
NEMO: NF-κB Essential Modulator
NLRP3: NOD-Like Receptor Family Pyrin Domain-Containing three
NLS: Nuclear Localization Signal
Nrf2: Nuclear Factor Erythroid 2-Related Factor 2
P2X7: P2X Purinoceptor 7
PAMPs: Pathogen-Associated Molecular Patterns
PI3K-Akt: Phosphoinositide 3-Kinase-Protein Kinase B
PK-PD: Pharmacokinetics-Pharmacodynamics
PRRs: Pattern Recognition Receptors
PYD: Pyrin Domain
p38 MAPK: p38 Mitogen-Activated Protein Kinase
ROS: Reactive Oxygen Species
RhoA/ROCK: Ras Homolog Gene Family, Member A/Rho-Associated Coiled-Coil Containing Protein Kinase
SAE: Sepsis-Associated Encephalopathy
SIRS: Systemic Inflammatory Response Syndrome
SOD: Superoxide Dismutase
TAK1: Transforming Growth Factor-β-Activated Kinase 1
TAD: Transactivation Domain
TEER: Transepithelial Electrical Resistance
TLR4: Toll-Like Receptor 4
TNF-α: Tumor Necrosis Factor-α
TRAF: TNF Receptor-Associated Factor
TCM: Traditional Chinese Medicine
Tregs: Regulatory T Cells
ATP: Adenosine Triphosphate
K+: Potassium Ion
Ca2+: Calcium Ion
